# INCREASED RISK OF COVID-19 INFECTION IN PEOPLE WITH DEPRESSION: EVIDENCE FROM ANALYSIS OF SHARE’S CORONA SURVEYS

**DOI:** 10.1093/geroni/igae098.2332

**Published:** 2024-12-31

**Authors:** Xiaona He, Wei Gao

**Affiliations:** Nanchang University, Nanchang, Jiangxi, China (People’s Republic); Nanchang University, Nanchang, Jiangxi, China (People’s Republic)

## Abstract

The mental disorder related COVID-19 infection remains of wide interest. However, there is a shortage of population-based studies on depression. To examine the association between a pre-existing depression and COVID-19 infection risk among middle-aged and older Europeans during the COVID-19 pandemic. A population-based retroprospective longitudinal study. Data were from the Survey of Health, Ageing and Retirement in Europe (SHARE), respondents 50 years and older from 26 European countries and Israel were included. Analysis was undertaken at the first and second years of the COVID-19 pandemic. Depression was assessd by the EURO-D scale. Outcomes were the COVID-19 infection during the period of COVID-19 pandemic. We used logistic regression to assess the association of pre-existing depression with the COVID-19 infection. Individuals who had a pre-existing depression had an increased risk for COVID-19 infection in SCS1 (OR=1.52, 95% CI: 1.28-1.79, P < 0.0001) and SCS2 (OR=1.24, 95% CI: 1.13-1.37, P < 0.0001). Among individuals with depression, obesity(BMI≥30) had higher risk than those with BMI≤30; OR=1.23, 95% CI: 1.03-1.46, P=0.021). Unvaccinated individuals with depression had higher risk than vaccinated individuals, with the strongest vaccination disparity for depression (OR=1.99, 95% CI: 1.65-2.39, P< 0.0001). Individuals with depression as being at increased risk for COVID-19 re-infection. (OR=1.68, 95% CI: 1.13-2.49, P< 0.0001). Depression may become an important factor affecting the population of middle-aged and older people infected with COVID-19, suggesting a need for more attention to the population. This includes providing mental health support and counseling services to reduce their symptoms of depression.

